# The impact of *de novo* liver metastasis on clinical outcome in patients with advanced non-small-cell lung cancer

**DOI:** 10.1371/journal.pone.0178676

**Published:** 2017-06-07

**Authors:** Yu-Ping Chang, Yu-Mu Chen, Chien-Hao Lai, Chiung-Yu Lin, Wen-Feng Fang, Cherng-Hua Huang, Shau-Hsuan Li, Hung-Chen Chen, Chin-Chou Wang, Meng-Chih Lin

**Affiliations:** 1 Division of Pulmonary and Critical Care Medicine, Department of Internal Medicine, Chang Gung Memorial Hospital Kaohsiung Medical Center, Chang Gung University College of Medicine, Kaohsiung, Taiwan; 2 Department of Respiratory Care, Chang Gung Institute of Technology, Chiayi, Taiwan; 3 Division of Hematology-Oncology, Department of Internal Medicine, Chang Gung Memorial Hospital Kaohsiung Medical Center, Chang Gung University College of Medicine, Kaohsiung, Taiwan; Seoul National University College of Pharmacy, REPUBLIC OF KOREA

## Abstract

Liver metastasis has been found to affect outcome in prostate cancer and colorectal cancer, but its role in lung cancer is unclear. The current study aimed to evaluate the impact of *de novo* liver metastasis (DLM) on stage IV non-small cell lung cancer (NSCLC) outcomes and to examine whether tyrosine kinase inhibitors (TKI) reverse poor prognosis in patients with DLM and epidermal growth factor receptor (EGFR)-mutant NSCLC. Among 1392 newly diagnosed NSCLC patients, 490 patients with stage IV disease treated between November 2010 and March 2014 at Kaohsiung Chang Gung Memorial Hospital were included. Patients were divided into two groups according to DLM status. There were 75 patients in the DLM group and 415 patients in the non-DLM group. The DLM group included more patients with bone metastasis, fewer patients with a lymphocyte-to-monocyte ratio (LMR) > 3.1, and fewer patients with pleural metastasis. In the DLM group, Eastern Cooperative Oncology Group performance status 3–4 and LMR ≦3.1 were associated with poor outcome. In patients without DLM, overall survival (OS) was longer in patients with EGFR-mutant NSCLC than in those without (20.2 vs. 7.3 months, p < 0.001). Among DLM patients, OS was similar between the EGFR-mutant and wild-type EGFR tumor subgroups (11.9 vs. 7.7 months, p = 0.155). We found that DLM was a significant poor prognostic factor in the EGFR-mutant patients treated with EGFR-TKIs, whereas DLM did not affect the prognosis of EGFR-wild-type patients.

## Introduction

In Taiwan and worldwide, lung cancer is the leading cause of cancer-related mortality [1]. About half of lung cancers are found at the advanced stage at diagnosis [2]. According to the lung cancer staging system of the American Joint Committee on Cancer (AJCC), 7th edition, lung to lung metastasis, pleural metastasis, and distant metastasis such as to brain, bone, and liver, among others, are classified as M1 disease and represent terminal stage cancer [3]. Median survival in patients with advanced lung cancer is usually 1 year or less [4], and patients with epidermal growth factor receptor *(EGFR)-*mutant metastatic non-small-cell lung cancer (NSCLC) may have longer overall survival (OS) when treated with tyrosine kinase inhibitors (TKIs) [5,6]. In the prediction of lung cancer survival, well-accepted prognostic factors are disease extent and Eastern Cooperative Oncology Group (ECOG) performance status (PS) [4]. Other predictors of survival such as extremes of age, carcinoembryonic antigen, *EGFR* mutation status, lymphocyte-to-monocyte ratio (LMR), number of metastatic sites, and hypoalbuminemia have also been proposed [4,7–13]. Therefore, even for cancers in the same stage, prognosis may be different. In castration-resistant prostate cancer, one study showed that patients with liver metastasis have shorter median OS [14]. Moreover, resection of liver metastasis in colorectal cancer was found to improve outcomes [15]. Thus, liver metastasis seem to play a role in the prognosis of both prostate cancer and colon cancer. However, no previous studies have examined their role in lung cancer outcomes. Therefore, we conducted a retrospective analysis to investigate the impact of liver metastasis on outcome in stage IV NSCLC patients. We also aimed to examine whether positive EGFR mutation status and first-line treatment with EGFR-TKIs reversed poor prognosis in stage IV NSCLC patients with *de novo* liver metastasis (DLM).

## Materials and methods

We retrospectively reviewed medical records of patients diagnosed with advanced NSCLC from November 2010 to March 2014 at Kaohsiung Chang Gung Memorial Hospital. Patients were included if they were over 18 years old and had confirmed stage IV NSCLC according to the AJCC 7^th^ edition criteria [3]. Lung cancer staging included chest computed tomography (CT); brain imaging (CT or magnetic resonance imaging); bone scans; pleural effusion cytology; and, in some cases, positron emission tomography. Data including basic information, metastatic site, progression-free survival (PFS), OS, and other related factors were collected and analyzed. PFS was defined as the period from the first day of treatment to documented disease progression, or death prior to disease progression. OS was defined as the period from the first day of treatment to death. Disease progression was determined according to Response Evaluation Criteria in Solid Tumors (RECIST) version 1.1 [16]. PS was defined based on ECOG criteria [17]. EGFR mutation analysis was performed using the Scorpion and amplified refractory mutation system (ARMS) techniques with formalin-fixed and paraffin-embedded tissue. DLM was defined as liver metastasis confirmed at the time of initial diagnosis.

Statistical analyses were performed using MedCalc (version14.10.2). PFS and OS were analyzed using Kaplan-Meier curves and log-rank testing. We used Cox proportional hazards regression models to evaluate independent factors that affected survival outcomes. Youden's index and receiver operating characteristic (ROC) curves were used to determine the best cutoff value of LMR. Comparisons of baseline clinical parameters between NSCLC patients with or without liver metastasis were made using the chi-square test or Fisher’s exact test for categorical variables and the unpaired t-test or Wilcoxon rank-sum test for continuous variables as appropriate. A p value < 0.05 was considered statistically significant.

The study was approved by the Institutional Review Board of Chang Gung Memorial Hospital, and the requirements for patient consent were waived (IRB:103-3226B).

## Results

### Patient and clinical characteristics

**Fig 1:**
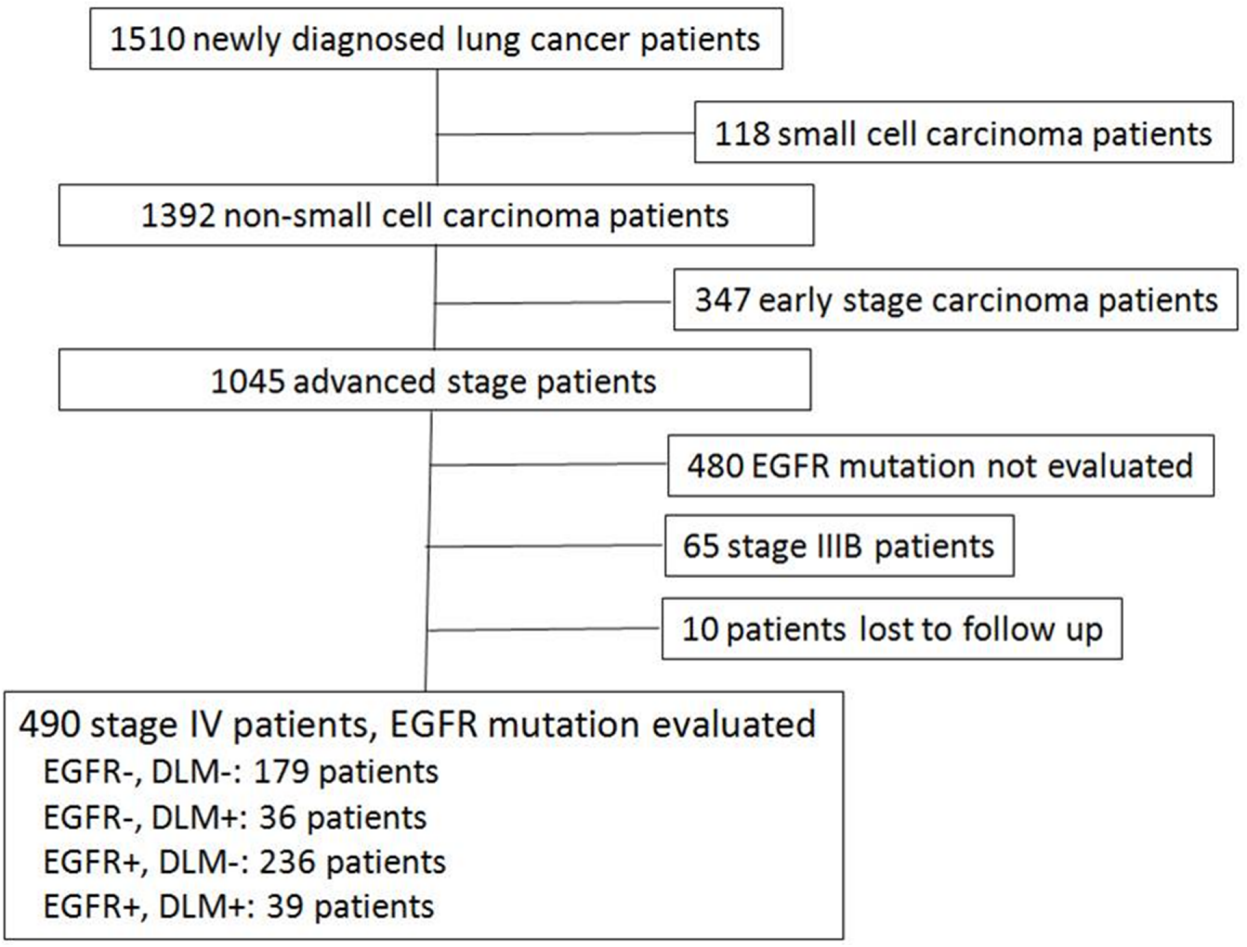
Inclusion and patient selection.

A total of 1510 patients received new diagnoses of lung cancer, and 1392 of these patients were diagnosed with NSCLC. Among these NSCLC patients, 490 patients with stage IV disease were included for evaluation, as shown in Fig 1. All EGFR-mutant patients received first-line treatment with TKIs, and patients with wild-type EGFR tumors received first-line chemotherapy or conservative treatment in cases of poor PS, according to clinician judgment. Mean age of all 490 patients was 63.8 ± 12.4 years. Basic data and clinical parameters are shown in Table 1.

**Table 1:** Comparison of baseline clinical parameters between NSCLC patients with or without liver metastasis.

	With *de novo* liver metastasis (n = 75)	Without *de novo* liver metastasis (n = 415)	*P* value
Age, years	61.8 ± 12.4	64.2 ± 12.3	0.130
BMI	22.5 ± 3.5	22.9 ± 3.9	0.428
Sex			0.062
Male	29 (38.7)	209 (50.4)	
Female	46 (61.3)	206 (49.6)	
DM			0.835
Yes	8 (10.7)	41 (9.9)	
No	67 (89.3)	374 (90.1)	
Smoking history			0.082
Never	54 (72.0)	255 (61.4)	
Former / current	21 (28.0)	160 (38.6)	
Performance status			0.443
ECOG 0–2	66 (88.0)	377 (90.8)	
ECOG 3–4	9 (12.0)	38 (9.2)	
EGFR mutation			0.435
Yes	39 (52.0)	236 (56.9)	
No	36 (48.0)	179 (43.1)	
Tumor type			0.758
Adenocarcinoma	65 (86.7)	354 (85.3)	
Non-adenocarcinoma	10 (13.3)	61 (14.7)	
LMR > 3.1	30 (40)	231 (55.7)	0.002
Brain metastasis	25 (33.3)	99 (23.9)	0.083
Bone metastasis	48 (64.0)	197 (47.5)	0.008
Pleural metastasis	22 (29.3)	179 (47.5)	0.025

BMI, body-mass index; DM, diabetes mellitus; ECOG, Eastern Cooperative Oncology Group; EGFR, epidermal growth factor receptor; LMR, lymphocyte-to-monocyte ratio; NSCLC, non-small-cell lung cancer.

Among the study patients, 75 patients had DLM, while 415 patients did not. There were no significant differences between these two groups in age, body mass index, sex distribution, presence of diabetes mellitus, smoking history, ECOG PS, EGFR mutation status, tumor type, or presence of brain metastasis. There were significantly fewer instances of LMR > 3.1, fewer cases of pleural metastasis, and more cases of bone metastasis in the DLM group. The best cutoff point for LMR determined by ROC curves was 3.1.

Further univariate and multivariate analysis of 490 NSCLC patients are shown in Table 2. BMI, sex, DM, ECOG PS, EGFR mutant status, tumor type, LMR, DLM were predictive factors in univariate analysis. Multivariate analysis showed that male gender, EOCG PS 3–4, without EGFR mutation, LMR ≤ 3.1, DLM were negative predictors for OS.

**Table 2:** Impact of baseline clinical parameters on NSCLC patients.

	Univariate analysis				Multivariate analysis		
	n	Event	OS (mo)	*p* value	Hazard ratio	*p* value	95% CI
Age, years							
>60	295	238	12.0	0.136			
≤ 60	195	154	14.4				
BMI							
>22	278	214	14.4	0.002	0.811	0.051	0.657–1.001
≤ 22	212	178	11.1				
Sex							
Male	238	197	11.9	0.005		0.012	0.618–0.943
Female	252	195	15.3		0.764		
DM							
Yes	49	46	9.3	0.015	1.172	0.332	0.851–1.613
No	441	346	13.4				
Smoking history							
Never	309	244	14.0	0.072			
Former/current	181	148	11.1				
Performance status							
ECOG 0–2	442	349	13.6	<0.001		0.001	1.281–2.520
ECOG 3–4	48	43	3.7		1.797		
EGFR mutation							
Yes	275	198	18.6	<0.001	0.569	<0.001	0.458–0.708
No	215	194	7.5				
Tumor type							
ADC	419	327	13.4	0.009		0.840	0.780–1.358
Non-ADC	71	65	10.2		1.029		
LMR							
>3.1	260	193	18.4	<0.001		<0.001	1.212–1.837
≤3.1	208	191	7.7		1.844		
DLM							
Yes	415	324	13.6	<0.001		0.01	1.092–1.880
No	75	68	8.8		1.432		

ADC, adenocarcinoma; BMI, body mass index; DM, diabetes mellitus; DLM, *de novo* liver metastases; ECOG, Eastern Cooperative Oncology Group; EGFR, epidermal growth factor receptor; LMR, lymphocyte-to-monocyte ratio; mo, months; NSCLC, non-small-cell lung cancer; OS, overall survival.

### Clinical characteristics of NSCLC patients with *de novo* liver metastasis

The results of univariate and multivariate analysis in the DLM group are shown in Table 3. ECOG PS 3–4 and LMR ≦ 3.1 were found to be associated with poor outcome, with hazard ratios of 1.5 and 7.4, respectively, in univariate analysis. Further analysis of these two parameters in multivariate analysis revealed hazard ratios of 6.83 (ECOG PS 3–4) and 2.10 (LMR ≦ 3.1). Extrahepatic metastasis were not found to affect outcome in univariate analysis in the DLM group.

**Table 3:** Impact of baseline clinical parameters on NSCLC patients with *de novo* liver metastasis.

	Univariate analysis				Multivariate analysis		
	n	Event	OS (mo)	*p* value	Hazard ratio	*p* value	95% CI
Age, years							
>60	34	30	8.8	0.555			
≤ 60	41	38	8.8				
BMI							
>22	41	36	11.4	0.166			
≤ 22	34	32	7.4				
Sex							
Male	29	27	8.3	0.901			
Female	46	41	9.0				
DM							
Yes	8	8	9.5	0.899			
No	67	60	8.8				
Smoking history							
Never	54	49	9.0	0.781			
Former/current	21	19	6.9				
Performance status							
ECOG 0–2	66	59	9.5	0.001		<0.001	2.478–18.802
ECOG 3–4	9	9	1.5		6.83		
EGFR mutation							
Yes	39	35	11.9	0.155			
No	36	33	7.7				
Tumor type							
ADC	65	58	8.7	0.325			
Non-ADC	10	10	8.8				
LMR							
>3.1	29	24	12.8	0.036		0.033	1.061–4.166
≤3.1	45	43	7.4		2.10		
Extrahepatic metastasis							
Yes	69	62	8.7	0.417			
No	6	6	17.1				

ADC, adenocarcinoma; BMI, body mass index; DM, diabetes mellitus; ECOG, Eastern Cooperative Oncology Group; EGFR, epidermal growth factor receptor; LMR, lymphocyte-to-monocyte ratio; mo, months; NSCLC, non-small-cell lung cancer; OS, overall survival.

### Impact of EGFR mutation status on patients with *de novo* liver metastasis

**Fig 2:**
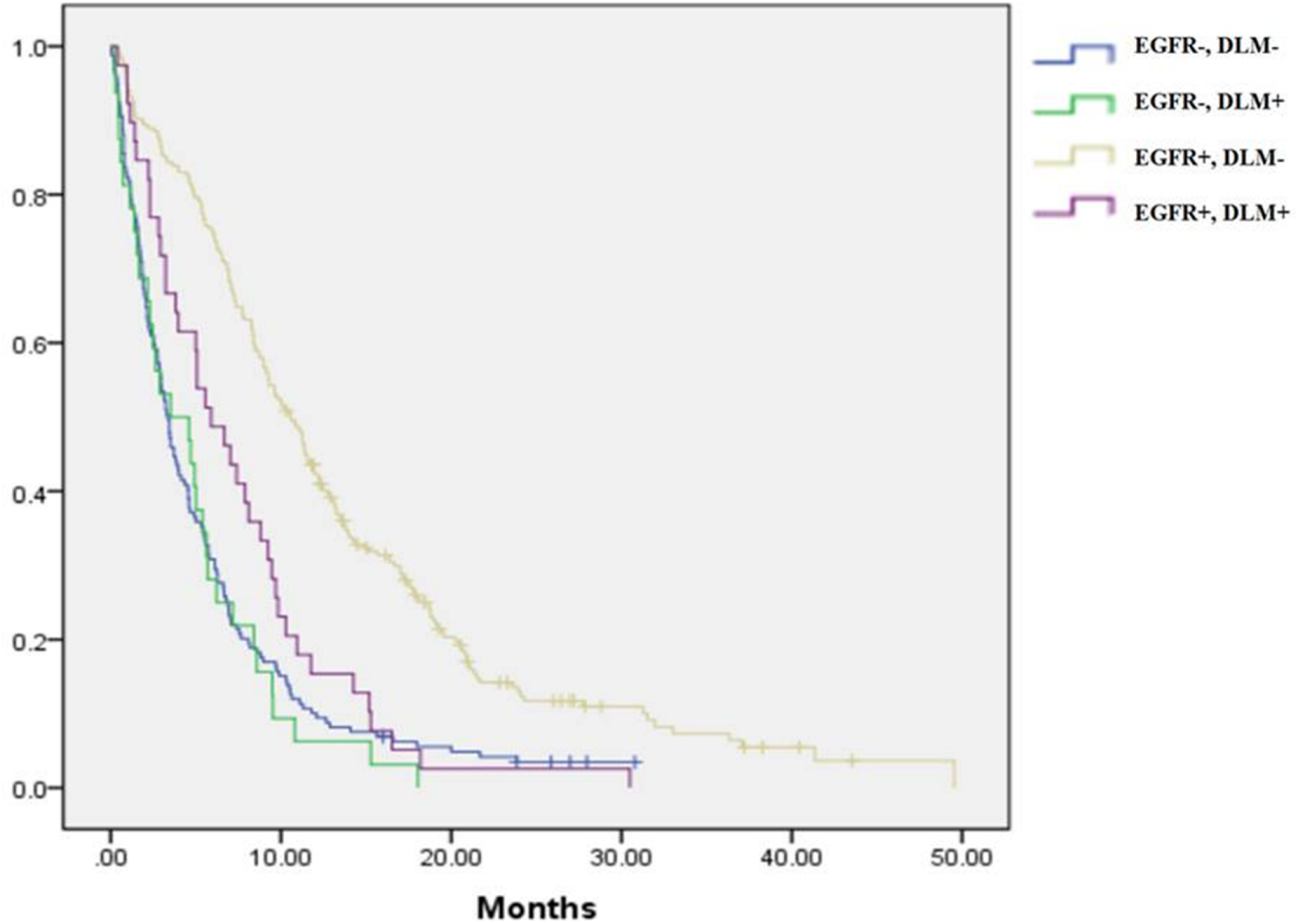
Progression-free survival regarding *de novo* liver metastasis and epidermal growth factor receptor mutation status.

**Fig 3:**
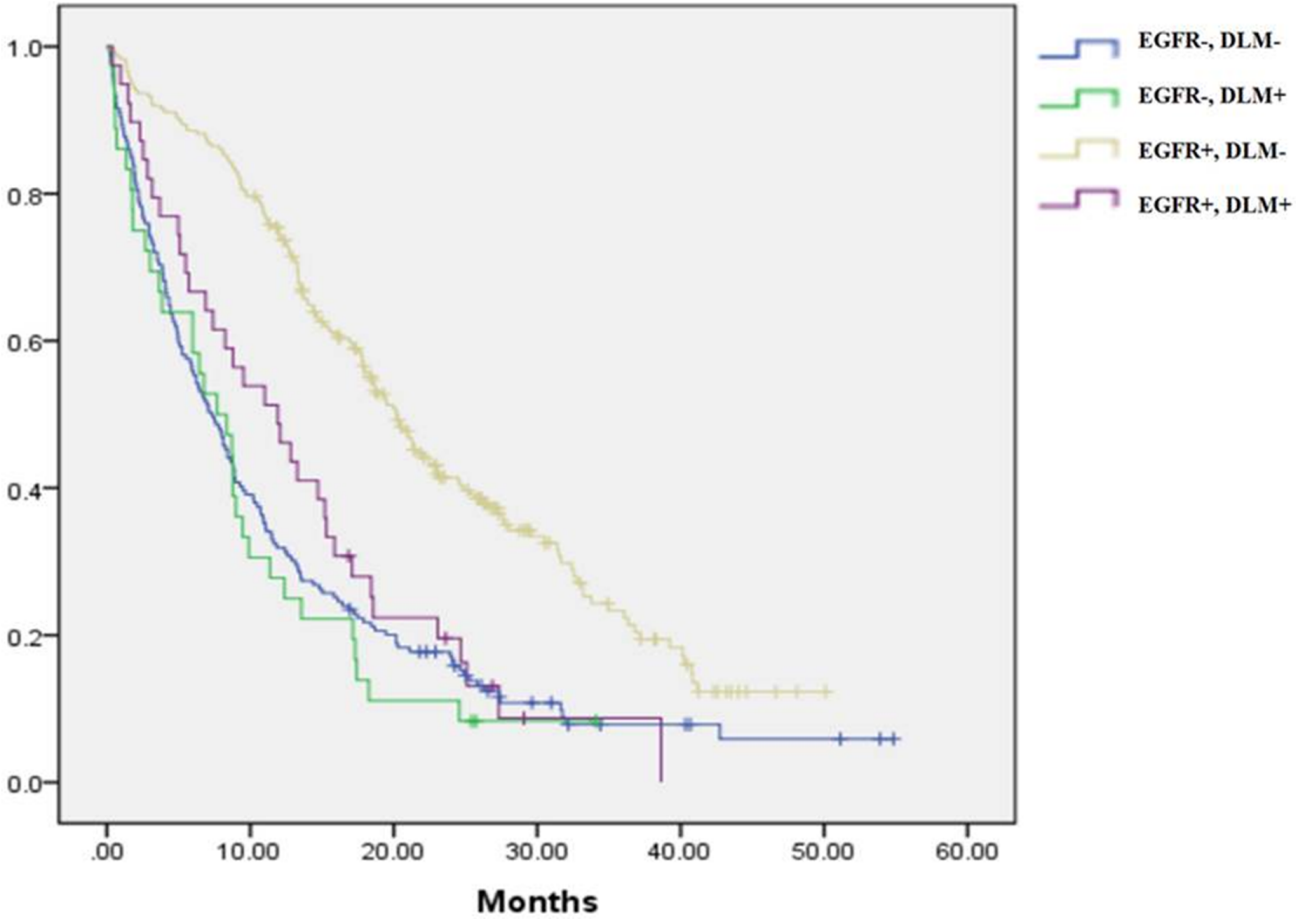
Overall survival regarding *de novo* liver metastasis and epidermal growth factor receptor mutation status.

Among patients with DLM, those who had EGFR mutation-positive disease and received EGFR-TKIs as first-line therapy had longer PFS than those with EGFR wild-type tumors (EGFR mutant vs. wild-type: PFS: 5.9 vs. 3.5 months, p = 0.046) (Figs 2 and 3). However, in patients with DLM, no significant OS benefit was observed in EGFR-mutant patients compared to those with EGFR wild-type disease in univariate analysis (EGFR mutant vs. wild-type: OS: 11.9 vs. 7.7 months, p = 0.155).

### Impact of EGFR mutation status on patients without *de novo* liver metastasis

In patients without DLM, those who had EGFR mutation-positive disease and received EGFR-TKIs as first-line therapy had longer PFS than those with EGFR wild-type tumors (EGFR mutant vs. wild type: PFS: 10.6 vs. 3.4 months, p < 0.001) (Figs 2 and 3). Furthermore, in patients without DLM, OS was longer in EGFR-mutant patients than in those with EGFR wild-type disease (EGFR mutant vs. wild-type: OS: 20.2 vs. 7.3 months, p < 0.001).

### Impact of DLM status on patients with EGFR-mutant and wild-type NSCLC

In patients with EGFR-mutant NSCLC, those with DLM had worse PFS and OS than those without DLM (DLM vs. non-DLM: PFS: 5.9 vs. 10.6 months, p < 0.001; OS: 11.9 vs. 20.2 months, p < 0.001). For those with EGFR wild-type NSCLC, the prognosis of non-DLM patients was no better than that of DLM patients (DLM vs. non-DLM: PFS: 3.5 vs. 3.4 months, p = 0.634; OS: 7.7 vs. 7.3 months, p = 0.521). It can be seen that the occurrence of DLM in patients with EGFR-mutant NSCLC results in outcomes as poor as those in patients with EGFR wild-type disease, with or without DLM.

## Discussion

The liver is a less common metastatic site of NSCLC than brain and bone, with an incidence of 47/72 (brain), 36/72 (bone), and 22/72 (liver) observed in the study by Quint et al [18,19]. Specific factors affecting metastasis to each particular site remain poorly understood. Hsu et al. found that among NSCLC patients, those with younger age and EGFR-mutant disease have a higher incidence of brain metastasis at initial diagnosis [20]. However, the authors did not present data regarding other concurrent metastatic sites. Chen et al. found that patients younger than 40 years of age were more likely to have brain, bone, liver, and pleural metastasis [21]. Furthermore, NSCLC patients with *ALK* gene rearrangement and EGFR mutations are more likely to have liver metastasis compared to patients without *ALK* gene rearrangement, EGFR mutation, and *KRAS* mutation [22]. Thus, EGFR mutation status and age appear to have some influence on brain or liver metastasis. However, in our study, age and prevalence of EGFR-mutant disease did not differ between the DLM and non-DLM groups. Furthermore, we observed an increased frequency of bone and pleural metastasis in the DLM group. In a study of 1074 patients with non-metastatic NSCLC treated with radiation therapy, the four most frequent sites of initial distant metastasis were brain (146/456), lung/effusion (125/456), bone (98/456), and liver (63/456) [23]. Thus, brain and bone seem more likely first sites of distant metastasis than liver. Accordingly, if a patient has liver metastasis, other concurrent distant metastasis may exist. This could partially explain our finding of a greater frequency of bone and pleural metastasis in the DLM group. The presence of liver metastasis may suggest more terminal status in stage IV NSCLC, since patients with liver metastasis tend to also have additional distant metastasis, and a greater number of metastatic sites predicts worse survival [4,8]. This could help explain why in our study, DLM patients were found to have worse prognosis than those in the non-DLM group. However, this question will require further study for confirmation.

**Fig 4:**
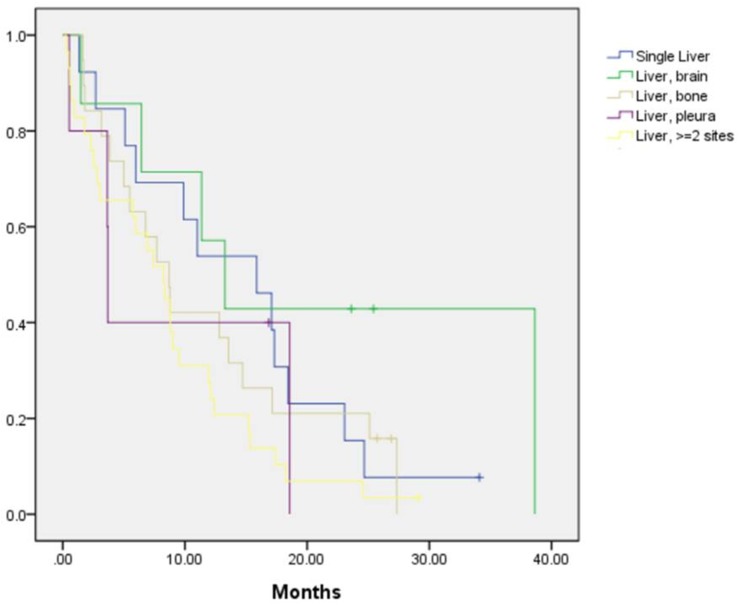
Overall survival of patients with liver plus other distant metastasis.

In the current study, the presence of EGFR mutations and first-line treatment with EGFR-TKIs were found to improve both PFS and OS in patients without DLM, as well as improve PFS in patients with DLM, but were associated with no improvement in OS in patients with DLM. A previous study by Vikram et al. showed that in stage IV NSCLC, brain, bone, and liver metastasis were not predictors of survival [8]. However, in the study by Hoang et al., liver metastasis was identified as a poor prognostic factor in patients with stage IIIB or IV NSCLC [4]. Furthermore, liver or bone metastasis as the first site of distant metastasis after radiation therapy for NSCLC was found to be associated with worse prognosis [23]. The results of these studies were not consistent, and the studies were performed in the era prior to EGFR mutation testing and TKIs. It has been demonstrated that TKIs improved PFS in EGFR-mutant NSCLC [24], and were also able to prolong OS [25]. The present study revealed that TKIs could improve both PFS and OS in patients without DLM, but in patients with DLM, administration of TKIs could not prolong OS even in cases of EGFR-mutant disease. A recent study conducted by Wu et al. showed that the presence of liver metastasis at initial diagnosis predicts poorer outcome in patients with stage IV EGFR-mutant lung adenocarcinoma treated with gefitinib as first-line therapy [26]. This result supports the findings of our study, as we demonstrated that among patients with EGFR-mutant disease, those with DLM had worse prognosis than those without DLM. However, the study by Wu et al. focused on EGFR-mutant adenocarcinoma. Our study further demonstrated that in patients with DLM, prognosis of EGFR-mutant group is as poor as wild-type group. The question of why treatment with TKIs was unable to reverse the poor prognosis in EGFR-mutant DLM patients will require further study to resolve. Comparison of OS of patients with single liver metastasis (n = 13), liver plus brain (n = 7), liver plus bone (n = 19), liver plus pleural (n = 5) metastasis, and liver plus more than 1 additional metastatic site (n = 29), as shown in Fig 4, reveals that there were no differences among the groups. Although the number of patients is small, it can be seen that the presence of liver metastasis leads to poor prognosis, regardless of the presence of other distant metastasis.

Our study has several limitations. First, it was retrospective in nature, and prospective studies are required to verify our findings. Second, the number of NSCLC patients with DLM was small in our study population. Whether a true negative result or inadequate power is the best explanation for the non-inferior outcomes of univariate analysis in NSCLC patients with DLM with or without EGFR mutation will require further investigation. Third, we didn’t check ALK gene rearrangement in all patients. Last, under-recognition of liver metastases is possible. As in Robinson’s study, the detection rate for lesion less than 1cm is 30–40% [27]. In Lardinois’ study, integrated positron-emission tomography (PET)-CT would be preferred approach for NSCLC staging [28], but in our study we mainly used chest CT, bone scan, brain magnetic resonance imaging for staging. Nevertheless, to the best of our knowledge, this is the first report discussing DLM in the era of TKIs. Maybe in the future, liver metastasectomy could be considered for NSCLC patients with liver metastases in order to improve survival.

## Conclusions

DLM was a significant poor prognostic factor in the EGFR-mutant patients treated with EGFR-TKIs, whereas DLM did not affect the prognosis of EGFR-wild-type patients.

## Supporting information

S1 Text Patient data. (XLS)
